# Ketamine induces opposite changes in AMPA receptor calcium permeability in the ventral tegmental area and nucleus accumbens

**DOI:** 10.1038/s41398-021-01658-3

**Published:** 2021-10-14

**Authors:** Olga Skiteva, Ning Yao, Karima Chergui

**Affiliations:** Molecular Neurophysiology Laboratory, Department of Physiology and Pharmacology, Karolinska Institutet, Stockholm, Sweden

**Keywords:** Depression, Molecular neuroscience

## Abstract

Ketamine elicits rapid and durable antidepressant actions in treatment-resistant patients with mood disorders such as major depressive disorder and bipolar depression. The mechanisms might involve the induction of metaplasticity in brain regions associated with reward-related behaviors, mood, and hedonic drive, particularly the ventral tegmental area (VTA) and the nucleus accumbens (NAc). We have examined if ketamine alters the insertion of the GluA2 subunit of AMPA receptors (AMPAR), which determines calcium permeability of the channel, at glutamatergic synapses onto dopamine (DA) neurons in the VTA and spiny projection neurons (SPNs) in the Core region of the NAc. Mice received one injection of either saline or a low dose of ketamine 24 h before electrophysiological recordings were performed. We found that GluA2-lacking calcium-permeable (CP) AMPARs were present in DA neurons in the VTA of mice treated with saline, and that ketamine-induced the removal of a fraction of these receptors. In NAc SPNs, ketamine induced the opposite change, i.e., GluA2-lacking CP-AMPARs were inserted at glutamatergic synapses. Ketamine-induced metaplasticity was independent of group I metabotropic glutamate receptors (mGluRs) because an agonist of these receptors had similar effects on glutamatergic transmission in mice treated with saline and in mice treated with ketamine in both VTA DA neurons and in the NAc. Thus, ketamine reduces the insertion of CP-AMPARs in VTA DA neurons and induces their insertion in the NAc. The mechanism by which ketamine elicits antidepressant actions might thus involve an alteration in the contribution of GluA2 to AMPARs thereby modulating synaptic plasticity in the mesolimbic circuit.

## Introduction

Ketamine elicits rapid and lasting antidepressant actions when administered in low doses to treatment-resistant patients with mood disorders such as major depressive disorder and bipolar depression [1, 2]. Ketamine’s antidepressant actions are thought to be mediated through the modulation of glutamatergic synaptic transmission and the induction of metaplasticity in brain regions involved in reward and motivation, important functions which are impaired in depressed patients. Ketamine was suggested to modify or induce long-term alterations in the strength of glutamatergic synaptic transmission, e.g., long-term potentiation (LTP) and long-term depression (LTD). Such types of synaptic plasticity might be altered in diseases and modified by a variety of pharmacological compounds. We previously demonstrated that ketamine prevented LTP induction in the Core area of the nucleus accumbens (NAc) [3]. This effect was observed one day following the injection and persisted for one week, demonstrating the ability of ketamine to induce lasting metaplasticity in the mesolimbic circuit. In dopamine neurons in the ventral tegmental area (VTA DA) from mice that received one injection of ketamine, responses mediated by AMPA receptors (AMPARs) were depressed. In these neurons, responses mediated by NMDA receptors (NMDARs) were unchanged, which caused a decreased AMPA/NMDA ratio, which is a measure of LTD. Loss of LTP in the NAc by a single injection of ketamine was associated with altered phosphorylation of the AMPAR GluA1 subunit. Contrasting with this observation in the NAc, LTD induced by ketamine in VTA DA neurons was not associated with a modified phosphorylation of GluA1. The precise mechanisms by which ketamine blunts increase in glutamatergic synaptic transmission in the NAc and promotes a novel form of LTD in VTA DA neurons remain to be resolved.

Ketamine-induced metaplasticity may be due to a change in the subunit composition of AMPARs and may thus modify the calcium permeability of these channels. Lack of the GluA2 subunit confers calcium permeability to AMPARs. GluA2-lacking, calcium-permeable (CP), AMPARs play important roles in synaptic plasticity, neuronal development, and might be involved in specific neurological diseases [4]. An altered calcium permeability of AMPARs due to a change in the insertion of GluA2 could underlie glutamatergic dysfunctions in mood disorders. Indeed, a decrease in GluA2 mRNAs was found in the entorhinal cortex of patients with bipolar disorder compared with controls [5]. Despite the known role of GluA2 in forms of synaptic plasticity and the ability of ketamine to induce metaplasticity, no electrophysiological studies have demonstrated insertion or removal of GluA2-containing, or GluA2-lacking, AMPARs from synapses following ketamine administration. Biochemical studies found that the total levels of GluA1 and GluA2 were increased in hippocampal, but not prefrontal cortex, synaptoneurosomes 24 h, but not 1 h, following ketamine administration [6]. Another study also found an increased surface expression levels of GluA1 and GluA2 in hippocampal slices, 3 h after ketamine administration to mice [7]. Our previous study raised the hypothesis that trafficking of AMPARs containing or lacking GluA2 might underlie the effects of ketamine at glutamatergic synapses in the mesolimbic DA system [3]. The NAc and VTA are implicated in reward-motivated behaviors, and GluA2-lacking CP-AMPARs receptors in these two brain regions might be involved in the mechanism of action of addictive drugs [8]. We therefore hypothesized that a change in the contribution of GluA2 to AMPARs at glutamatergic synapses in the mesolimbic DA system might contribute to the antidepressant mechanism of action of ketamine. The aim of the present study was to determine if a low dose of ketamine impacts the incorporation of GluA2 to AMPARs at glutamatergic synapses onto VTA DA neurons and in spiny projection neurons (SPNs) in the Core area of the NAc.

## Materials and methods

### Animals, treatments, and brain slice preparation

The experimental procedures were authorized by our local ethical committee (Stockholms norra djurförsöksetiska nämnd) and were described previously [3]. Male C57BL/6 J mice aged 5−11 weeks were purchased from Envigo (The Netherlands). We did not use female mice because our previous study, which was done with males [3], did not investigate the effect of ketamine on synaptic transmission and plasticity in the VTA and NAc in female animals. Mice had free access to water and food and were kept on a 12 h−12 h light-dark cycle. Mice were administered a single i.p. injection of either vehicle (saline) or ketamine (10 mg kg^−1^) as done in our previous study [3]. No randomization was performed to allocate mice in different groups. The experimenters were not blinded to the injections because the persons who injected the mice with saline or ketamine also performed the electrophysiological experiments. Twenty-four hours after the injection, mice were anesthetized with isoflurane, their brains were rapidly removed and submerged in a slicing solution containing (in mM): glucose (10), NaH_2_PO_4_ (1), KCl (2), NaCl (15.9), sucrose (219.7), MgCl_2_ (5.2), CaCl_2_ (1.1), and NaHCO_3_ (26). Coronal hemisections (200−400 μm thick) containing the midbrain and the NAc were obtained using a microslicer (VT 1000 S, Leica Microsystem, Heppenheim, Germany). The sections were incubated in a modified artificial cerebrospinal fluid (aCSF) containing (in mM): glucose (10), NaH_2_PO_4_ (1.2), NaHCO_3_ (23.4), KCl (2.5), NaCl (126), MgCl_2_ (4.7), and CaCl_2_ (1) at 32 °C for 1 h after the slicing and then at 28 °C.

### Brain slice electrophysiology

**Fig. 1:**
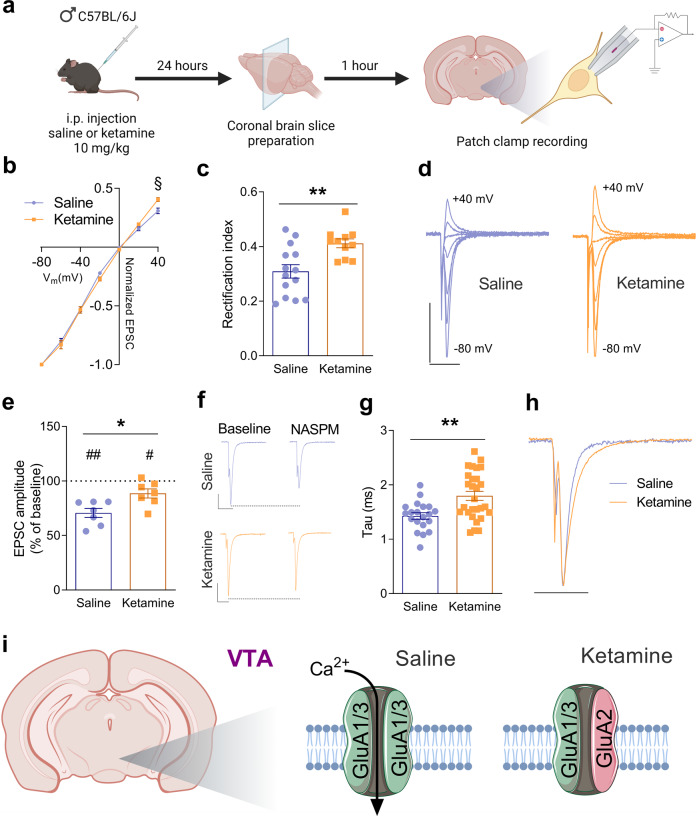
Ketamine promotes the insertion of calcium impermeable AMPARs at glutamatergic synapses onto VTA DA neurons. **a** Schema illustrating the experimental design. **b** I/V relationships of AMPAR-EPSCs amplitude normalized by −80mV in VTA DA neurons from saline- and ketamine-treated mice. §*P* = 0.0262, two-way ANOVA. **c** Rectification index from the neurons in (**a**) Saline: 0.31 ± 0.025 (*n* = 14 neurons from 4 mice), ketamine: 0.41 ± 0.016 (*n* = 11 neurons from 5 mice). **P = 0.0032 unpaired *t*-test. **d** Example traces of AMPAR-EPSCs recorded at holding potentials between −80 mV and +40 mV with 20 mV increments in a VTA DA neuron from a saline-treated mouse and in a VTA DA neuron from a ketamine-treated mouse. Scale bars represent 200 pA and 10 ms for both neurons. **e** Effect of bath application of NASPM (25 μM), a GluA2-lacking CP-AMPAR blocker; % of baseline in saline-treated mice: 70.67 ± 4.2 (*n* = 7 neurons from 4 mice), and in ketamine-treated mice: 88.56 ± 4.2 (*n* = 7 neurons from 4 mice). *P = 0.0107 unpaired *t*-test. ^#^*P* = 0.0317, ^##^*P* = 0.001 paired *t*-test. **f** Example traces of AMPAR-EPSCs before (baseline) and during the application of NASPM in the perfusion solution in a VTA DA neuron from a saline-treated mouse and in a VTA DA neuron from a ketamine-treated mouse. Scale bars represent 100 pA and 10 ms for both neurons. **g** AMPAR-EPSC tau (ms) in saline-treated mice: 1.43 ± 0.06 (*n* = 20 neurons from 7 mice), and in ketamine-treated mice: 1.8 ± 0.08 (*n* = 28 neurons from 10 mice). **P = 0.0014 unpaired *t*-test. **h** Superimposed AMPAR-EPSCs traces illustrating the slower kinetics in a VTA DA neuron from a ketamine-treated mouse compared to that recorded in a VTA DA neuron from a saline-treated mouse. Scale bar represents 10 ms for both neurons. **i** Schema summarizing the effect of ketamine on the involvement of GluA2 in synaptic AMPARs in VTA DA neurons.

**Fig. 2:**
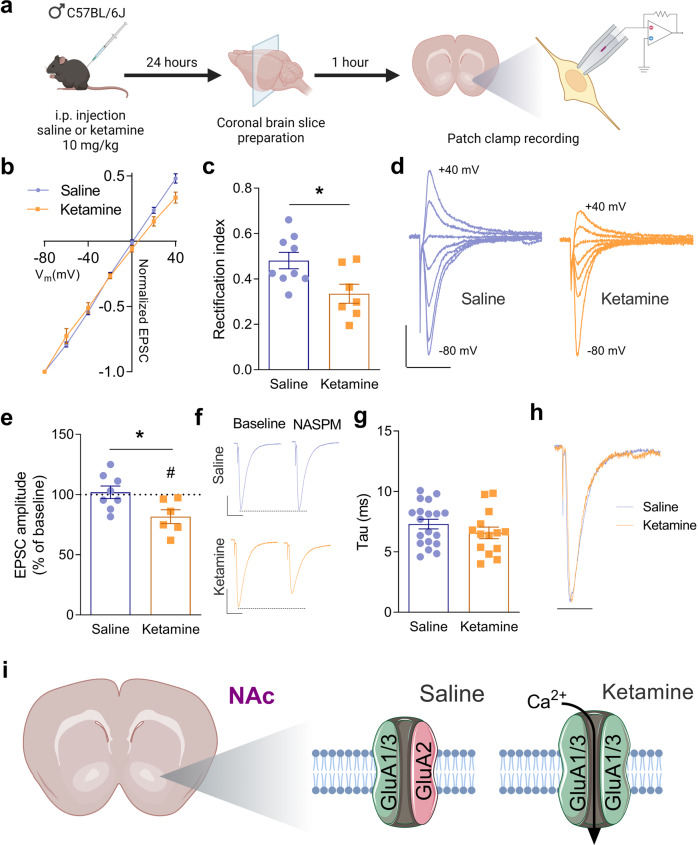
Ketamine promotes the insertion of CP-AMPARs at glutamatergic synapses on NAc SPNs. **a** Schema illustrating the experimental design. **b** I/V relationships of AMPAR-EPSCs amplitude normalized by −80mV in NAc SPNs from saline- and ketamine-treated mice. **c** Rectification index from the neurons in (**a**) Saline: 0.481 ± 0.04 (*n* = 9 neurons from 5 mice), Ketamine: 0.335 ± 0.04 (*n* = 7 neurons from 3 mice). *P = 0.0197 unpaired *t*-test. **d** Example traces of AMPAR-EPSCs recorded at holding potentials between −80 mV and +40 mV with 20 mV increments in a NAc SPN from a saline-treated mouse and in a NAc SPN from a ketamine-treated mouse. Scale bars represent 100 pA and 20 ms for both neurons. **e** Effect of bath application of NASPM (25 μM); % of baseline in saline-treated mice: 101.9 ± 5.2 (*n* = 8 neurons from 4 mice), and in ketamine-treated mice: 81.6 ± 5.7 (*n* = 6 neurons from 4 mice), *P = 0.0228 unpaired *t*-test. ^#^*P* = 0.0237 paired *t*-test. **f** Example traces of AMPAR-EPSCs before (baseline) and during the application of NASPM in the perfusion solution in a NAc SPN from a saline-treated mouse and in a NAc SPN from a ketamine-treated mouse. Scale bars represent 100 pA and 20 ms for both neurons. **g** AMPAR-EPSC tau (ms) in saline-treated mice: 7.3 ± 0.4 (*n* = 19 neurons from 5 mice), and in ketamine-treated mice: 6.57 ± 0.5 (*n* = 14 neurons from 4 mice), *P* = 0.2499 unpaired *t*-test. **h** Superimposed AMPAR-EPSCs traces illustrating similar kinetics in a NAc SPN from a ketamine-treated mouse and in a NAc SPN from a saline-treated mouse. Scale bar represents 20 ms for both neurons. **i** Schema summarizing the effect of ketamine on the involvement of GluA2 in synaptic AMPARs in NAc SPNs.

We performed whole-cell patch-clamp recordings of VTA DA neurons and SPNs in the Core area of the NAc as done previously [3, 9]. Neurons were identified as being DA or SPNs based on several anatomical, morphological, and electrophysiological criteria described in earlier reports [10, 11]. DA neurons were located in the VTA, had a large soma size, a slow spontaneous firing (<6 Hz), displayed an Ih current and membrane capacitance >40 pF. Our previous study verified that the neurons that we recorded in the VTA contained tyrosine hydroxylase [3]. SPNs were located in the Core area of the NAc, ventral to the anterior commissure, had a small soma size, no spontaneous firing, an input resistance of around 100 MΩ and resting membrane potential around −85 mV. These criteria allowed us to differentiate between DA neurons/SPNs and non-DA neurons in the VTA/interneurons in the NAc. Cells that did not meet the criteria for VTA DA neurons or SPNs were not further recorded and were not included in the analyses. Patch electrodes (3–5 MΩ) were filled with a solution containing (in mM): 140 CsCl, 2 MgCl_2_, 1 CaCl_2_, 10 HEPES, 10 EGTA, 2 MgATP, 0.3 Na_3_GTP, pH adjusted to 7.3 with CsOH. Excitatory postsynaptic currents (EPSCs) mediated by AMPA receptors (AMPARs) were measured at −80 mV, in the whole-cell mode, in the presence of gabazine (SR-95531, 10 μM), an antagonist of GABA_A_ receptors. DL-AP5 (50 μM) was used in some of the recordings (e.g., I/V relationships) to block NMDA receptors. EPSCs were induced by electrical stimulations via a patch pipette filled with aCSF placed close to the recorded neuron. We used a glass electrode filled with aCSF, positioned on the slice surface in the Core area of the NAc, to record extracellular field potentials. We used a stimulating electrode (concentric bipolar, FHC, Bowdoinham, ME) placed in the slice, near the recording electrode, to evoke field excitatory postsynaptic potentials/population spikes (fEPSP/PSs). Stimulation pulses were applied every 15 s to the brain slice through the stimulation electrode. Single stimuli were applied at an intensity yielding 50−60% maximal response as assessed by a stimulus/response curve established, for each slice, at the beginning of the recording session, by increasing the stimulation intensity and measuring the amplitude of the fEPSP/PS. Signals were amplified via a GeneClamp 500B or an Axopatch 700B amplifier (Axon Instruments, Foster City, CA), acquired at 10 kHz, and filtered at 2 kHz. We used the pClamp 10 software (Axon Instruments, Foster City CA, USA) to acquire and analyze data. The schema of our experimental design is presented in Fig. 1a and Fig. 2a.

### Chemicals and drugs

We used the following compounds for slice electrophysiology (all from Hello Bio, Bristol, UK): Gabazine/SR95531 (Cat. No. HB0901), 1-napthyl acetyl spermine trihydrochloride (NASPM) (Cat. No. HB0441), DL-AP5 (Cat. No. HB0251), and (R,S)−3,5-DHPG (Cat. No. HB0026). These compounds were prepared in stock solutions, diluted in aCSF to their final concentration (10 μM for gabazine; 25 μM for NASPM; 50 μM for DL-AP5 and 20 or 100 μM for DHPG) and applied in the recording chamber through the perfusion solution. (±)-ketamine hydrochloride was obtained from Sigma-Aldrich, Stockholm, Sweden (Cat. No. K2753).

### Statistical analysis

The GraphPad Prism 8 software was used for data analysis and statistics. Most of our data have equal variance, as determined using the test of equal variance in the GraphPad Prism software. We used the Kolmogorov-Smirnov test to assess the normal distribution of the data. No sample size calculations were made. No test for outliers has been applied and no data points were excluded. Data are expressed as mean ± s.e.m. The numbers of neurons or slices examined, and the numbers of mice used are indicated in the Figure legends. We used the Student-*t* test for unpaired and paired observations to assess the statistical significance of the results. For I/V curves, we used a repeated measures (RM) two-way ANOVA test with Greenhouse-Geisser correction followed by Sidak test for multiple comparisons. Significant levels were set at *P* < 0.05.

## Results

### Ketamine induces the removal of a fraction of GluA2-lacking CP-AMPARs at glutamatergic synapses in VTA DA neurons

To investigate possible changes induced by ketamine in the involvement of GluA2 in synaptic AMPARs in VTA DA neurons, we first performed current/voltage (I/V) relationship analyses of AMPAR-EPSCs. We then examined the effect of NASPM trihydrochloride, a selective antagonist of GluA2-lacking CP-AMPARs, on the amplitude of AMPAR-EPSCs. In saline-treated mice, I/V relationships showed rectification at positive membrane voltages, while in ketamine-treated mice I/V relationships rectified less and were more linear (Fig. 1b, d). The rectification index (ratio between EPSC amplitude at +40 mV and that at −80 mV) was significantly increased in ketamine-treated mice (Fig. 1c). In saline-treated mice, NASPM (25 μM) decreased the amplitude of AMPAR-EPSCs (Fig. 1e, f), demonstrating the presence of GluA2-lacking CP-AMPARs at glutamatergic synapses onto VTA DA neurons. In ketamine-treated mice, NASPM also decreased the amplitude of AMPAR-EPSCs, but this decrease was significantly reduced when compared to saline-treated mice (Fig. 1e, f). This reduced effect of NASPM, together with the changes in the I/V curve and rectification index, suggests that ketamine induces the removal of a fraction of GluA2-lacking CP-AMPARs and promotes the insertion of GluA2-containing, calcium impermeable, AMPARs in VTA DA neurons (Fig. 1i). In addition to the presence or absence of GluA2, RNA editing of this subunit at the Q/R site affects the properties of the channel by reducing conductance and calcium permeability [12]. Furthermore, the different editing statuses of GluA2 confer different kinetics of AMPAR-mediated responses [13]. We analyzed the kinetics of AMPAR-EPSCs in VTA DA neurons by measuring the decay time constant (Tau). We found a significantly slower EPSC in ketamine- than in saline-treated mice (Fig. 1 g, h), suggesting that ketamine might induce a change in GluA2 editing.

### Ketamine promotes the insertion of GluA2-lacking CP-AMPARs at glutamatergic synapses in NAc SPNs

We performed the same experiments described for VTA DA neurons in NAc SPNs to investigate the possibility that ketamine induces metaplasticity in these neurons by altering the contribution of GluA2 to synaptic AMPARs (Fig. 2a). I/V relationships of AMPAR-EPSCs in NAc SPNs of saline-treated mice were linear but a rectification was observed in ketamine-treated mice (Fig. 2 b, d). A two-way ANOVA test, which examines responses at all voltages, did not reveal any statistically significant differences between saline- and ketamine-treated mice. However, the rectification index was significantly decreased in ketamine-treated mice, suggesting a change in the contribution of GluA2 to synaptic AMPARs (Fig. 2c). To test this possibility, we used the selective antagonist of GluA2-lacking CP-AMPARs, NASPM. NASPM (25 μM) did not decrease the amplitude of the AMPAR-EPSC in NAc SPNs from saline-treated mice (Fig. 2 e, f). This result demonstrates that GluA2-lacking CP-AMPARs are not present at these synapses. In NAc SPNs from ketamine-treated mice, however, NASPM decreased the amplitude of the AMPAR-EPSC (Fig. 2e, f), demonstrating the insertion of GluA2-lacking CP-AMPARs (Fig. 2 i). The decay time constants of AMPAR-EPSCs were comparable in saline-treated mice and in ketamine-treated mice (Fig. 2g, h), suggesting an unaltered GluA2 editing in NAc SPNs.

### Ketamine does not induce metaplasticity through group I mGluRs

**Fig. 3:**
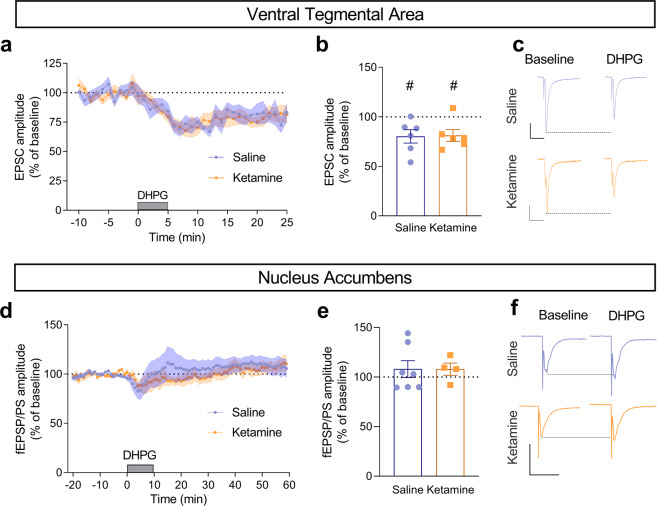
Effect of DHPG on glutamatergic synaptic transmission is not altered by ketamine. **a**, **d** Time course of the effect of bath application of DHPG (**a**: 20 μM; **b:** 100 μM), a group I mGluR agonist, on the amplitude of AMPAR-EPSCs in VTA DA neurons (**a**) and fEPSP/PS in the NAc (**d**) in saline- and ketamine-treated mice. **b**, **e** Percent of baseline in VTA DA neurons (**b**): saline 80.3 ± 6.8 (*n* = 6 neurons from 4 mice), ketamine 81.2 ± 5.9 (*n* = 6 neurons from 3 mice). ^#^*P* = 0.0332 and 0.0252 paired *t*-test. *P* = 0.9169 unpaired *t*-test. Percent of baseline in the NAc (**e**): saline 108.0 ± 8.6 (*n* = 7 slices from 4 mice), ketamine 107.9 ± 6.2 (*n* = 4 slices from 3 mice), *P* = 0.9917 unpaired *t*-test. **c**, **f** Example traces of AMPAR-EPSCs before (baseline) and during the application of DHPG in the perfusion solution in VTA DA neurons (**c**). Scale bars: 100 pA/20 ms for the neuron from a saline-treated mouse and 50 pA/20 ms for the neuron from a ketamine-treated mouse. Example traces of fEPSP/PSs in the NAc before (baseline) and during the application of DHPG (**f**). Scale bars: 1 mV/10 ms for both slices.

At glutamatergic synapse onto VTA DA neurons, stimulation of group I metabotropic glutamate receptors (mGluRs) triggers a form of LTD which is associated with incorporation of GluA2-containing AMPARs [14]. Given that ketamine induces a form of LTD [3] and promotes the insertion of GluA2-containing AMPARs in VTA DA neurons, it is possible that ketamine-LTD uses the same cellular mechanisms as group I mGluR-LTD. To test this possibility, we assessed the ability of a group I mGluR agonist, DHPG, to induce LTD in VTA DA neurons and in the NAc. We found that DHPG induced an LTD in VTA DA neurons in saline- and in ketamine-treated mice with no statistical difference between the two groups (Fig. 3a-c). A form of LTD induced by group I mGluRs activation was also described in the NAc [15, 16]. In the present study, DHPG did not modify glutamatergic synaptic transmission in the NAc of saline-treated mice (Fig. 3d-f), and thus failed to induce LTD. In the NAc of ketamine-treated mice, DHPG also failed to induce LTD (Fig. 3d-f).

## Discussion

We found that a low dose of ketamine causes lasting changes in the contribution of GluA2 to synaptic AMPARs, and thus in calcium permeability, in the mesolimbic circuit. These changes are opposite in VTA DA neurons and in NAc SPNs. Ketamine-induced metaplasticity at glutamatergic synapses onto VTA DA neurons involves removal of a fraction of GluA2-lacking CP-AMPARs. In NAc SPNs, ketamine promotes the insertion of GluA2-lacking CP-AMPARs. These results contribute to a better understanding of the role of AMPARs in the mesolimbic circuit in the therapeutic versus side effects of ketamine.

The antidepressant action of ketamine is suggested to be mediated through the modulation of AMPARs in the prefrontal cortex, hippocampus, and mesolimbic circuit [6, 17, 18]. However, a limited number of reports have investigated the effect of ketamine on the expression of GluA2 in the brain. One day after ketamine treatment, the levels of GluA2 were shown to be increased in the postsynaptic density from the medial prefrontal cortex and decreased in the hippocampus [19]. In contrast to these findings, in another study, ketamine increased the levels of GluA2 in hippocampal, but not prefrontal cortex, synaptoneurosomes 24 h after the injection, likely contributing to the increased glutamatergic synaptic strength in the hippocampus [6]. Unfortunately, no electrophysiological data are available to support a change in the contribution of GluA2 to AMPARs at glutamatergic synapses in the brain following treatment with ketamine. In the present study, we found that ketamine induces opposite changes in the contribution of GluA2 to synaptic AMPARs in the VTA and NAc. In VTA DA neurons, we demonstrate that I/V relationships rectify less in ketamine-treated mice than in saline-treated mice. Given that GluA2-lacking CP-AMPARs display non-linear I/V relationships due to inward rectification [20], these results indicate that ketamine reduces AMPAR calcium permeability by promoting the contribution of GluA2 to synaptic AMPARs. This was confirmed by the reduced effect of NASPM on the amplitude of AMPARs in VTA DA neurons from ketamine-treated mice. Thus, ketamine induces the removal of a fraction of GluA2-lacking CP-AMPARs and promotes the insertion of GluA2-containing, calcium impermeable, AMPARs. Our previous study demonstrated that ketamine induces a novel form of LTD in VTA DA neurons which were not associated with an altered phosphorylation of the GluA1 subunit of AMPARs [3]. Ketamine-induced LTD was demonstrated by a reduced AMPA/NMDA ratio, and reduced amplitude of spontaneous EPSCs (sEPSCs). Our present results demonstrate reduced AMPAR calcium permeability in VTA DA neurons from ketamine-treated mice. In addition, we found that AMPAR-EPSCs have slower decay time constants in VTA DA neurons from ketamine-treated mice as compared to saline-treated mice. Furthermore, the observation that a group I mGluR agonist, DHPG, induces LTD in VTA DA neurons from both saline- and ketamine-treated mice suggests that ketamine-LTD does not occlude group I mGluR-LTD. Thus, the mechanisms of induction of ketamine-LTD do not involve the activation of group I mGluRs. Together, these results demonstrate that the mechanism by which ketamine induces a novel form of LTD at glutamatergic synapses onto VTA DA neurons involves the insertion of GluA2-containing, calcium impermeable, AMPARs, and an altered RNA editing at sites including the Q/R site in GluA2.

Our previous study demonstrated that ketamine induces metaplasticity in the NAc [3]. Thus, ketamine blunts increases in glutamatergic synaptic transmission and inhibits LTP induction through a mechanism that likely involves an altered GluA1 phosphorylation. In the present study, we found that Group I mGluRs are unlikely to be involved in metaplasticity induced by ketamine in the NAc. Indeed, ketamine treatment does not affect the lack of modulatory effect on glutamatergic synaptic transmission by DHPG. The inability of DHPG to induce plasticity is possibly due to the age of the mice used in the present study as we described that DHPG-LTD is induced in adolescent mice, but not adult mice [16]. Our findings demonstrate that ketamine promotes the removal of GluA2-containing, calcium-impermeable, AMPARs, and the insertion of AMPARs with high conductance, lacking GluA2. These receptors might contribute to the ability of ketamine to prevent potentiation of glutamatergic synapses when glutamatergic inputs are strongly activated, and counter a loss of synaptic depression, as suggested earlier [3].

The suitability of ketamine for the treatment of depression in a large scale is limited because of its abuse potential, dissociative and psychotic properties [1, 21, 22]. Interestingly, addictive drugs, including cocaine and amphetamine, induce LTP, block LTD, and promote the insertion of GluA2-lacking CP-AMPARs at glutamatergic synapses onto VTA DA neurons [8, 23]. Ketamine causes the opposite, i.e., it replaces CP-AMPARs from synapses with calcium impermeable AMPARs and induces a form of LTD. These changes at glutamatergic synapses in VTA DA neurons might therefore underlie the antidepressant action of ketamine rather than its abuse potential. In the NAc, insertion of GluA2-lacking CP-AMPARs occurs after around 6 weeks of withdrawal from cocaine self-administration and was suggested to mediate the incubation of cocaine craving [24]. It remains to be investigated if ketamine-induced side effects are through the insertion of CP-AMPARs in NAc SPNs. Several studies implicate AMPARs as pharmacological targets for the development of effective treatments of depression [22, 25]. The present study further suggests GluA2 as a potential novel target for the treatment of mood disorders.
